# Molecular Analysis of Caprine Enterovirus Circulating in China during 2016–2021: Evolutionary Significance

**DOI:** 10.3390/v14051051

**Published:** 2022-05-15

**Authors:** Xiaoran Chang, Qian Lin, Qun Zhang, Junying Hu, Gulbahar Tursun, Yingrui Deng, Chunguang Guo, Xinping Wang

**Affiliations:** State Key Laboratory for Zoonotic Diseases, Key Laboratory of Zoonosis Research of Ministry of Education, College of Veterinary Medicine, Jilin University, Changchun 130012, China; changxr20@mails.jlu.edu.cn (X.C.); carolynxiaoxiao@163.com (Q.L.); zhangqun21@mails.jlu.edu.cn (Q.Z.); jyhu19@mails.jlu.edu.cn (J.H.); glbhe19@mails.jlu.edu.cn (G.T.); Emilydyr0228@gmail.com (Y.D.); cxr970114@163.com (C.G.)

**Keywords:** caprine/ovine enterovirus, CEV-JL14, phylogenetic analysis, genotype, evolution, alignment analysis

## Abstract

Here, we report the characterization of 13 novel caprine/ovine enterovirus strains isolated from different regions in China during 2016–2021. Immunoperoxidase monolayer assay showed that these viral strains shared strong cross-reaction with the previously reported caprine enterovirus CEV-JL14. Alignment analysis of the complete nucleotide sequences revealed 79.2%–87.8% and 75.0%–76.7% sequence identity of these novel caprine enterovirus strains to CEV-JL14 and TB4-OEV, respectively. Phylogenetic analyses clustered these novel strains to EV-G based on the amino acid sequences of P1 and 2C+3CD. Moreover, phylogenetic analysis of these caprine enterovirus strains identified three new EV-G types using VP1 sequences. These results demonstrate the genetic variations and the evolution of caprine enterovirus.

## 1. Introduction

Enteroviruses (EV) are a large group of viruses that have a significant impact on human and animal health. As members of the genus of *Enterovirus* within the family *Picornaviridae*, they cause infectious diseases characterized by digestive, respiratory, and nervous disorders, such as poliomyelitis, coxsackievirus infection, echovirus infection and hand, foot, and mouth disease (HFMD). According to the latest viral classification, the *Enterovirus* genus is divided into 12 species of enteroviruses (A, B, C, D, E, F, G, H, I, J, K, and L) and 3 species of rhinoviruses (A, B, and C) [1]. Out of the 12 enterovirus species, EV-E and EV-F viruses infect cattle [2,3,4,5,6,7], while EV-G viruses generally infect pigs [8,9,10,11]. Recently, several enterovirus strains isolated from goats or sheep were also classified as EV-G [12,13,14,15]. Chang et al. reported the caprine enterovirus strain SD-S67 isolated in cell culture from samples collected from goats as being EV-F, suggesting that goats are also natural hosts for EV-F [16]. Currently, there are 20 types within EV-G, including 2 types (EV-G5 and EV-G7) from sheep, 1 type from goats (EV-G20), and 17 from pigs or wild boar [1].

Enteroviruses are single-stranded positive-sense RNA viruses with an icosahedral symmetry and no envelope. The genome size of enteroviruses varies from 7100 to 7500 nucleotides, containing a single large open reading frame (ORF) flanked by 5′ and 3′ untranslated regions (UTRs) [1,17]. The 5′ UTR contains an internal ribosome entry site (IRES) responsible for the initiation of translation [18], while the 3′ UTR contains a sequence of 100–180 bp followed by a poly A tail of a different length. Currently, the 5′ UTR sequence is usually used as one of the important regions for enterovirus classification [19]. The ORF encodes a polyprotein that is initially processed into P1, P2, and P3 during translation or post-translation. The precursor P1 protein is further proteolytically cleaved to generate the viral structural proteins VP1, VP2, VP3, and VP4, which constitute the basic structure of the virus, while precursor P2 and P3 are proteolytically cleaved to produce the nonstructural proteins 2A, 2B, 2C, 3A, 3B, 3C, and 3D, which play an important role in the process of viral infection and replication [17,20]. The capsids of enteroviruses are composed of four structural proteins: VP1, VP2, VP3, and VP4. The shell is formed by VP1, VP2, and VP3, with VP4 lying on its inner surface. According to the crystal structure analysis, the structural protein VP1 is the most exposed part of the virion. This protein constitutes two important structures: the “canyons” and “pocket factors”. The depressions around the quintet of the icosahedron are called “canyons”, while the hydrophobic “pocket factors” are hidden inside the pore and stabilize the virions [21]. VP1 contains six surface loops (BC, DE, BE/aB, GF, GH, and HI), which are mainly located around the icosahedral pentad, exposed on the surface of the virion, and are the main variable region of the virus [22].

Caprine/ovine enterovirus (CEV/OEV) infection has been increasingly reported in recent years [12,13,14,15]. However, the genetic variation and evolution of caprine enterovirus has scarcely been investigated. Currently, the complete genome sequences of caprine/ovine enterovirus have been revealed only in very few strains [14]. The majority of the reported sequences of caprine/ovine enterovirus are either partial 5′ UTRs or a small portion of the coding sequence, such as VP1 genes [13,15]. Therefore, it is difficult to evaluate the genetic variation and evolution of caprine enterovirus, especially under the circumstance of the paucity of complete genome sequences. Previously, we reported the isolation of the first caprine enterovirus strain CEV-JL14 from goats with severe diarrhea, and showed that CEV-JL14 had just above 77.1% nucleotide sequence identity to the ovine enterovirus strain TB4-OEV [14]. Sequence analysis clustered CEV-JL14 strain as a new type (EV-G20) in EV-G [1,11,14]. Originally, serotypes were defined by their antigenic and cross-neutralization properties. However, it has since been demonstrated that different enterovirus serotypes consistently show greater than 25% nucleotide sequence divergence from one another in the *VP1* gene, while variants of the same serotype show less than 25% divergence [1]. Newly characterized enteroviruses and rhinoviruses have been classified based on genetic relationships, and information on their antigenic characteristics is often not available. Simmonds et al. recommend that the term “type” rather than “serotype” be used for the nomenclature of all enteroviruses and rhinoviruses [23]. Here, we expand our report of the new isolates from different regions in China during 2016–2021, unveil the complete genome sequence, and explore the genetic variation and evolutionary patterns of the caprine enterovirus.

## 2. Materials and Methods

### 2.1. Cell Culture and Viral Isolation

**Figure 1:**
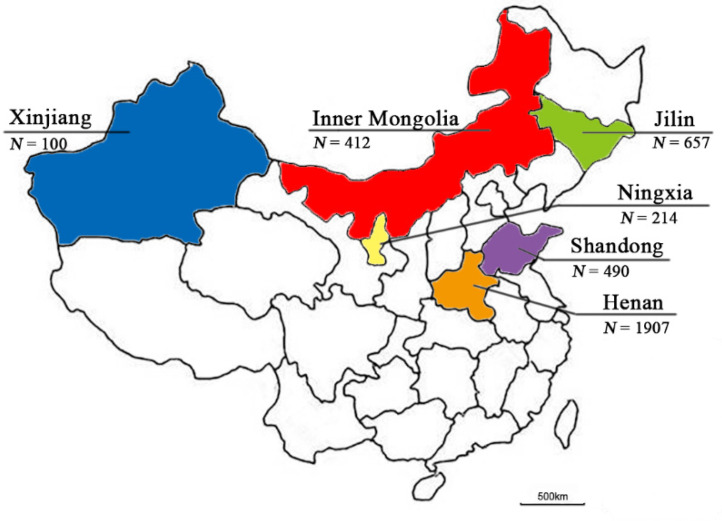
Geographic locations for specimen collection: The different provinces are marked in different colors with the number of collected samples. Blue: Xinjiang; red: Inner Mongolia; green: Jilin; yellow: Ningxia; purple: Shandong; orange: Henan. *N* represents the number of samples collected.

Vero cells were cultured in Dulbecco’s modified Eagle’s medium (DMEM) (Invitrogen, Carlsbad, CA, USA) supplemented with 5% fetal bovine serum (HyClone, Beijing, China), 2 µg/mL gentamycin, and 2 mM L-glutamine (Invitrogen). A total of 3780 fecal swabs collected from a total of 106 goat farms in Jilin, Shandong, Ningxia, Henan, Inner Mongolia, and Xinjiang was processed as previously described [7], and used to isolate the virus using Vero cells. The geographic region for sample collections in China is illustrated in Figure 1. The cells were observed for potential cytopathic effect (CPE) every 6 h post-inoculation. CPEs were captured using a Canon digital camera (Canon, Tokyo, Japan). The non-infected cells were used as negative controls.

### 2.2. Viral Characterization

Characterization of the viral strains was performed following a standard procedure. TCID50 was determined following the procedure previously described in [7]. Briefly, the 5th generation of the harvested virus was 10-fold serially diluted and used to inoculate the 96-well cell culture plate with quadruplicate repeats for each dilution. CPEs were observed and counted within 48–72 h post-inoculation (hpi). TCID50 viral titers were calculated following the Reed–Muench method [24]. The non-infected cells were used as negative controls. 

### 2.3. Immunoperoxidase Monolayer Assay (IPMA)

IPMA was performed as previously described in [25]. Briefly, Vero cells cultured in 24-well plates were infected with the isolated viruses. Eight hours post-inoculation, the cells were washed with PBS, fixed with ice-cold methanol for 20 min at −20 °C, and probed with a monoclonal antibody (mAb) against CEV-JL14-VP1 (1:500) for 1 h at 37 °C. After washing, the cells were further incubated with HRP-conjugated goat anti-mouse IgG antibody (1:1000, Sigma, St. Louis, MO, USA) for 45 min at 37 °C. The plates were then stained with 3-amino-9-ethylcarbazole (Amresco, Olympia, WA, USA) substrate and visualized using the Canon digital camera (Canon, Tokyo, Japan). Cells infected with the CEV-JL14 virus were used as positive controls. Non-infected cells were used as negative controls. 

### 2.4. RNA Extraction, cDNA Synthesis, and PCR Amplification

RNA extraction was performed using TRIzol^TM^ reagent (Invitrogen, Carlsbad, CA, USA) following the manufacturer’s instructions, as previously described in [7]. cDNA synthesis was carried out using the Bio RT-cDNA kit (Invitrogen), following the manufacturer’s instructions. PCR amplification of the complete genome sequence was performed in a total volume of 50 μL of reaction system containing 25 μL of the premixed Taq polymerase, 2 μL of synthesized cDNA, 1 μM of each primer, and ddH_2_O. The primers used for amplification of the complete genome sequence were designed based on the variation of each strain (Table S1). PCR was performed following a program of denaturing the reaction mixture at 95 °C for 5 min, followed by 35 cycles of denaturation at 95 °C for 30 s, annealing at 56 °C for 30 s, and extension at 72 °C for 30 s, with extra final extension for 10 min at 72 °C. cDNA ends were determined using the FirstChoice RLM-RACE kit (Thermos Fisher Scientific, Carlsbad, CA). PCR-amplified products were either directly sent out for sequencing or cloned to T-vectors (Promega, Madison, WI, USA) before sequencing (Sangon Biotechnology, Shanghai, China). The resulting sequences served as a template for searching for homologous sequences in GenBank (www.ncbi.nlm.nih.gov, 25 September 2021). The complete genome sequences were assembled using Lasergene software (version 7.0, DNASTAR, Madison, WI, USA).

### 2.5. Bioinformatics Analysis 

Alignment analysis of multiple sequences was performed using the Clustal W method [26]. Briefly, the P1, 2C+3CD, and VP1 amino acid sequences for these isolated caprine/ovine enterovirus strains were analyzed with the representative sequences of all known types in EV-G and the representative sequences of all 15 species of the *Enterovirus* genus as outgroup sequences, respectively. The complete genomic sequences of the reference strains are available in the GenBank database, and the accession number information is listed in Table S2. Phylogenetic analysis was initially performed using neighbor-joining (NJ) methods [27] and Jones–Taylor–Thornton substitution models with a bootstrap value of 1000 to generate the phylogenetic tree [28]. Pairwise distances (p-distances) of the aligned enterovirus VP1 sequences were calculated using MEGA-7 software [29]. Recombination analysis was performed with SimPlot software using the Hamming model, with a window size of 200 nt and a step size of 20 nt [16,27].

## 3. Results

### 3.1. Isolation and Characterization of the Enterovirus Strains from Goat/Sheep Herds

Previously, we reported the identification of the first caprine enterovirus strain CEV-JL14 [14]. Later, this strain was designated as type EV-G20 [11]. To examine the genetic variation and evolution of caprine enterovirus, fecal samples were collected from goat/sheep herds in different provinces characterized by manifestations of diarrhea during 2016–2021 in China. CEV-positive specimens detected by ELISA were processed to isolate the potential viruses [30]. Thirteen caprine/ovine virus strains were isolated from goat/sheep farms from various regions in China (Figure 1). The related information on these caprine/ovine virus strains is listed in Table S3. Typical CPEs of enterovirus were observed in cells infected with JL-LS34, JL-LS127, JL-LS165, XJ-287, XJ-274, XJ-259, HeN-T3-12, HeN-D2-57, and SD-S68 strains in the 1st passage, while CPEs were only observed in JL-LS174, NMG-F37, HeN-D1-37, and NX-DR26 strains after the 3rd blind passage. All viral strains used for future characterization were in the 5th passage. As detailed in Table S3, TCID_50_ titers for the novel viral strains were determined, ranging from 10^3^/0.1 mL to 10^6.5^/0.1 mL. Surprisingly, the TCID_50_ titer for the strain JL-LS174 was only 10^3^/0.1 mL, which is significantly lower than those of the strains JL-LS34, JL-LS127, and JL-LS165 from the same goat herds. 

### 3.2. Immune Cross-Reaction of the Novel Caprine Enterovirus Strains to CEV-JL14

**Figure 2:**
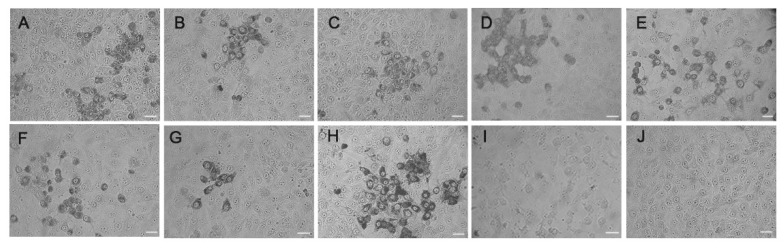
Immunoreactions of novel caprine/ovine enterovirus isolates to CEV-JL14. Seven representative novel caprine/ovine enterovirus strains were characterized by IPMA. Immunoreactions of the isolated viral strains to a monoclonal antibody against CEV-JL14-VP1 are shown. Strong reactions were revealed in the cells infected with NX-DR26, NMG-F37, SD-S68, JL-LS34, JL-LS127, JL-LS165, JL-LS174, and CEV-JL14 (**A**–**H,** respectively), while no reaction was observed for SD-S67 virus and normal Vero cells (**I**,**J**). Panels A–I represent the isolates NX-DR26, NMG-F37, SD-S68, JL-LS34, JL-LS127, JL-LS165, JL-LS174, CEV-JL14, and SD-S67, respectively. J represents the non-infected cell control. Bar = 20 μm.

To determine whether these caprine/ovine virus strains with full-length genome sequences shared any immune cross-reaction with the CEV-JL14 virus, IPMA assay was performed using the monoclonal antibody against VP1 encoded by the CEV-JL14 virus [14,16]. Vero cells infected with CEV-JL14 (EV-G) and SD-S67 (EV-F) were used as positive and negative controls, respectively. As shown in Figure 2, when antibodies against the CEV-JL14 virus were incubated with NX-DR26, NMG-F37, SD-S68, JL-LS34, JL-LS127, JL-LS165, JL-LS174, and CEV-JL14, the immunoreactions were observed for these viral strains in comparison with the normal-cell control, suggesting that these caprine enterovirus strains shared common antigenicity with the CEV-JL14 virus. It was also observed that caprine enterovirus SD-S67 (Figure 2I)—an EV-F strain—had no cross-reaction with the CEV-JL14 strain. 

### 3.3. Unveiling of the Complete Genome Sequences of Caprine/Ovine Enterovirus

To determine the complete sequences of the novel viral strains, a series of primers were designed individually for each strain and used to amplify the genomic sequences. After sequencing and joining the overlapped PCR-amplified fragments, the complete genome sequences for these viruses were obtained. Sequence analyses showed that the lengths of complete genome sequences for these novel strains varied from 7447 to 7469 nt. While the lengths of the 3′UTR were relatively diverse, ranging from 106 nt to 137 nt, the lengths of the 5′UTR were relatively conserved, ranging from 819 nt to 822 nt. Furthermore, the ORFs for these novel strains were highly conserved, ranging from 6513 nt to 6519 nt, and encoding a polyprotein of 2171 aa–2173 aa. In addition, all strains shared a typical picornavirus genome organization. The newly identified viral strains and their GenBank accession numbers are listed in Table S4.

### 3.4. High Sequence Identity Shared by These New Caprine/Ovine Enterovirus Strains with CEV-JL14

To determine the sequence identity of these novel caprine/ovine enterovirus strains with the reference strains—including caprine enterovirus CEV-JL14, and the ovine enteroviruses TB4-OEV, 990/IK-NI, and EV-F SD-S67—sequence alignment analyses on the complete genome sequences of these viral strains were initially performed. As shown in Table 1, these seven strains shared a relatively high nucleotide sequence identity with the CEV-JL14 strain, varying from 79.2% to 87.8%, while they shared a nucleotide sequence identity of 75.0%𢀓76.7% with TB4-OEV. The VP1 amino acid sequence identity of the novel caprine/ovine enterovirus strains with CEV-JL14 and TB4-OEV was 64.2%–95.8% and 62.8%–74.3%, respectively (Table 2). Further sequence analysis revealed a relatively higher sequence identity among these new caprine enterovirus strains. These results indicate that the newly identified caprine enterovirus strains are more closely related to the caprine enterovirus CEV-JL14 strain than the ovine enterovirus TB4-OEV, and that they evolved dramatically in relation to CEV-JL14. 

### 3.5. Novel Caprine/Ovine Enterovirus Strains Phylogenetically Similar to EV-G, and Three New EV-G Types Revealed

**Figure 3:**
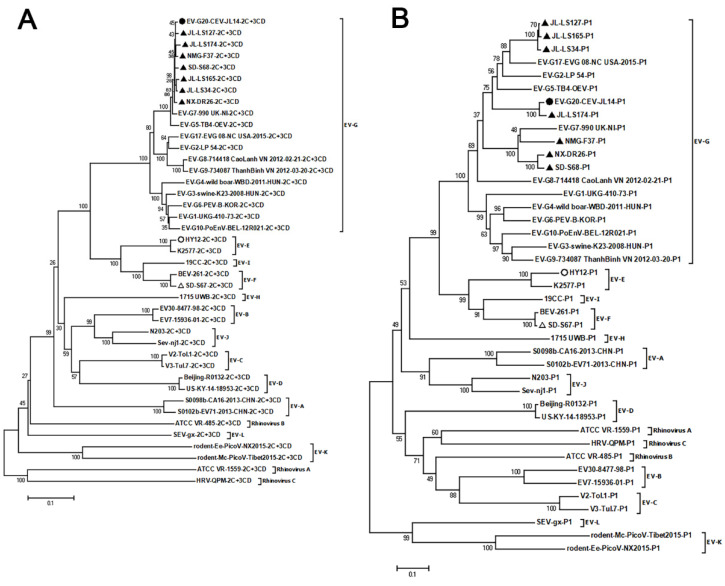
Phylogenetic analyses for the novel caprine/ovine enterovirus strains. The reference sequences include the representative sequences of all known EV-G types and representative sequences of all 15 species of the *Enterovirus* genus as outgroup sequences. The amino acid sequences of 2C+3CD (**A**) and P1 (**B**) were used to construct the phylogenetic tree using the NJ method with 1000 bootstrap replications. Bootstrap values of > 50 are shown at the nodes. The scale bar represents 10% nucleotide sequence divergence for NJ methods. Viruses are marked with symbols as follows: ▲ refers to the strains obtained in this study from goats and sheep; ● stands for the CEV-JL14 strain; ○ refers to the HY12 EV-E strain isolated from cattle; △ stands for the SD-S67 strain (an EV-F) isolated from goats.

To determine the status of the novel caprine/ovine enteroviruses, phylogenetic analysis was performed based on the amino acid sequences of 2C+3CD and P1, using the NJ method, with representative sequences of all known types of EV-G and representative sequences of all 15 species of the *Enterovirus* genus as outgroup sequences. Since no 2C+3D or P1 sequences were available in NCBI for EV-G11-EV-G16, EV-G18, and EV-G19, these EV-G types were excluded in the 2C+3D and P1 phylogenetic tree analyses. As shown in Figure 3A,B, all seven caprine/ovine enterovirus strains were clustered in the same clade as EV-G, making them different from these of EV-E and EV-F, indicating that these caprine/ovine strains belong to the EV-G type. 

**Figure 4:**
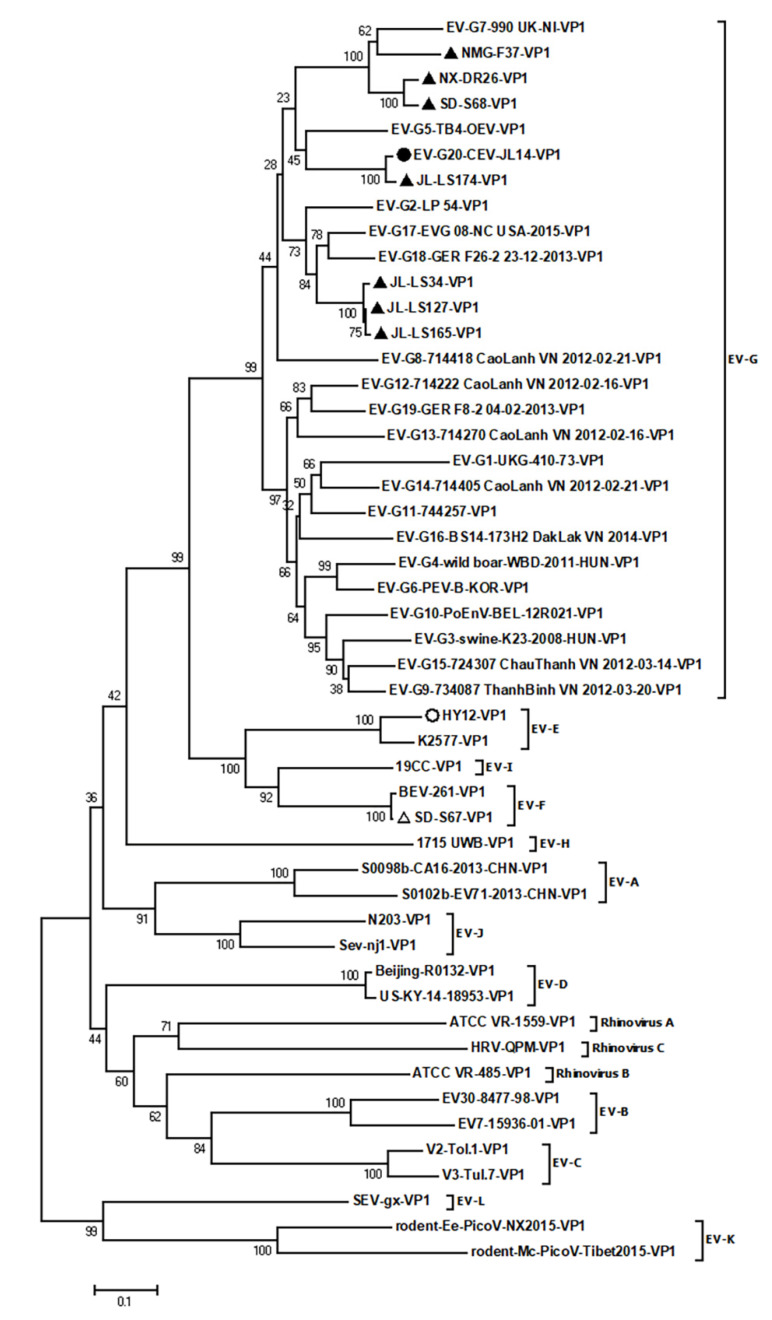
Three novel EV-G types revealed in caprine/ovine enterovirus: Phylogenetic analysis was performed based on the VP1 amino acid sequences. The reference sequences include the representative sequences of all known EV-G types and representative sequences of all 15 species of the *Enterovirus* genus as outgroup sequences. The phylogenetic tree was generated using the NJ method with 1000 bootstrap replications. Bootstrap values of > 50 are shown at the nodes. The scale bar represents 10% nucleotide sequence divergence for NJ methods. Viruses are marked with symbols as follows: ▲ refers to the strains obtained in this study from goats and sheep; ● stands for the CEV-JL14 strain; ○ refers to the HY12 EV-E strain isolated from cattle; △ stands for the SD-S67 strain (an EV-F) isolated from goats.

Further phylogenetic analysis revealed three new types for the new isolated caprine/ovine enterovirus based on the sequence identity of VP1 (Figure 4). The first type contained the strains JL-LS34, JL-LS127, and JL-LS165, which had amino acid sequence identity of 97.9%–99.3% (Table 2) amongst themselves. The p-distance from the VP1 amino acid sequences of these three strains to the EV-G reference strains was 0.19–0.38 (Table S5), while the p-distance between themselves was 0.01–0.02 (blue). The VP1 amino acid sequence identity of these three strains with EV-G was 59.3%–74.3% (Table 2), suggesting that JL-LS34, JL-LS127, and JL-LS165 belong to a new type, designated as EV-G21. The second type consisted of the strains NX-DR26 and SD-S68, which had 95.1% (Table 2) amino acid sequence identities, with a p-distance of 0.04 (orange in Table S5). The VP1 sequence identity of these two strains with EV-G was 65.2%–75.3% (Table 2), with a p-distance of 0.23–0.47 (Table S5), indicating that NX-DR26 and SD-S68 also belong to a new type, designated as EV-G22. The third type contained the strain NMG-F37, whose amino acid sequence identity with EV-G was less than 77.4% (Table 2), with a p-distance of 0.21–0.42 (Table S5), indicating that NMG-F37 belongs to another new type, named EV-G23. The strain JL-LS174 was clustered to the same type as CEV-JL14 (EV-G20), where they shared 95.8% (Table 2) amino acid sequence identity, with a p-distance of 0.04 (Table S5).

**Figure 5:**
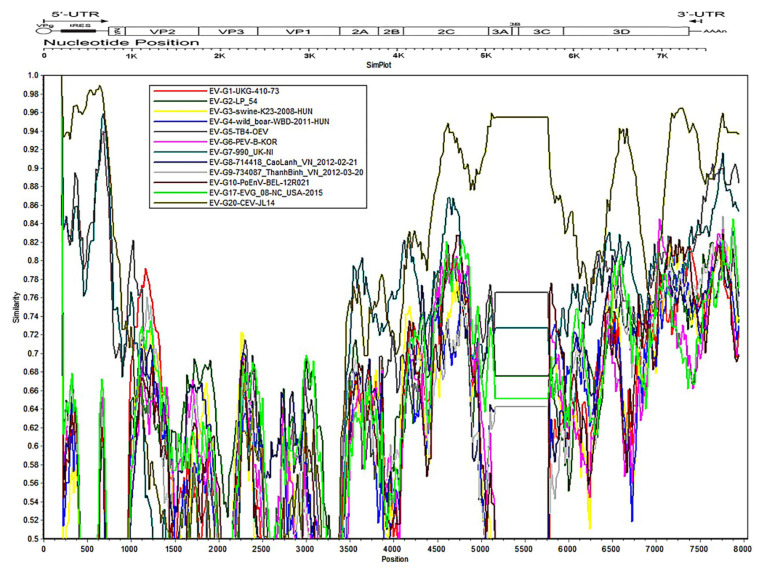
Recombination analyses of JL-LS34 with EV-G. Each curve is a comparison between the genome being analyzed and a reference genome. The horizontal bar above the curves is an illustration of the enterovirus genome. Each point plotted is the percentage identity within a sliding window 200 nt wide centered on the position plotted, with a step size of 20 nt between points.

To explore whether the isolated strains harbored the sequences from other EV-G viruses, recombination analyses were performed for these isolated caprine/ovine enterovirus strains, using SimPlot software. As shown in Figure 5, the nonstructural protein portion of JL-LS34 had the highest homology to CEV-JL14, and no apparent recombinations with EV-G were found. Similar results were also observed in the JL-LS127 (Figure S1), JL-LS165 (Figure S2), NX-DR26 (Figure S4), SD-S68 (Figure S5), and NMG-F37 (Figure S6) strains. Although the JL-LS174 strain had the highest homology with CEV-JL14 in terms of the complete genome sequence, no apparent recombination events with EV-G were observed in this strain (Figure S3).

## 4. Discussion

Caprine/ovine enterovirus is an emerging virus revealed in recent years that is associated with severe or mild digestive and respiratory disease. Although the complete genome sequence of two ovine and one caprine enterovirus has been uncovered, the genetic variation and evolution of the caprine enterovirus remains unknown due to the paucity of the complete genome sequences. Previously, we reported the isolation of a caprine enterovirus CEV-JL14 from goats with severe diarrhea. After sequence analysis, CEV-JL14 was designated as EV-G20—a new type in EV-G [1,11]. To expand our findings, we further isolated 13 novel caprine/ovine enterovirus isolates from the major goat-raising regions during 2016–2021. Results from IPMA assays showed an immune cross-reaction between those newly isolated caprine enteroviruses and the first caprine enterovirus strain CEV-JL14, suggesting that they are closely related to CEV-JL14. 

Phylogenetic analysis clustered the caprine enterovirus into a distinct clade consisting of caprine/ovine enterovirus strains, which is different from enterovirus strains of porcine origin within EV-G. The diversity or the genetic variation of the capsid proteins determines the viral type at the molecular level, and the amino acid sequence identity of VP1 is currently considered the standard for enterovirus classification [31]. Zell et al. suggested that enteroviruses be classified based on the amino acid sequence identities of the VP1 capsid protein, where 70% to 85% sequence identity was used for heterologous types and >90% for homologous types [19]. Recently, several studies have also proposed criteria for enterovirus type based on the identities of the VP1 nucleotide or amino acid sequences, where nucleotide sequence identities of <75% and amino acid sequence identities of <88% were grouped in the same types for heterologous types of homologous species [32,33]. Sequence analyses for the caprine enterovirus demonstrate the diversity of VP1 amino acid sequence identity among the caprine/ovine enteroviruses. Based on the criteria for enterovirus type, we propose that the available caprine enterovirus strains should be divided into six different types. In addition to the current EV-G5, EV-G7, and EV-G20, three novel types within EV-G were proposed. Since the JL-LS34, JL-LS127, and JL-LS165 strains were grouped in a branch with 97.9% amino acid sequence identity, they were proposed as the novel type EV-G21. The JL-LS174 virus shared a 95.8% amino acid sequence identity with CEV-JL14, and was grouped in the existing type EV-G20. As the NX-DR26 and SD-S68 strains had 84.1% nucleotide sequence and 95.1% amino acid sequence identities, and the VP1 amino acid sequence identity of these two strains with EV-G was 65.2%–75.3%, they were designated as the new type EV-G22. Furthermore, the NMG-F37 strain had an amino acid sequence identity of less than 77.4% with EV-G, indicating that it is another new type, suggested as EV-G23. The results of division of the caprine enterovirus strains into six types demonstrated the diversity of caprine enteroviruses and the genetic variation and evolution of caprine/ovine enteroviruses. The unveiling of different types existing in the same herd indicates the possibility that the JL-LS174 strain might have originated from different herds, and then later been introduced and mixed with various populations. Since goats are traded and circulated widely between different regions in China, the unveiling of two types in the same herd indicates that the JL-LS174 strain (an EV-G20 strain) might have originated from a different herd and then later introduced to the herd infected with the proposed EV-G21 type (JL-LS34, JL-LS127, and JL-LS165). Additionally, our results showed that NX-DR26 and SD-S68—two caprine enterovirus isolates from different regions—were clustered in the same genotype, suggesting that this type is also likely a dominant type circulating in the goat herds in China, which is also a subject for future investigation.

In summary, we characterized seven novel caprine/ovine enterovirus strains and revealed the diversity and genetic variation of caprine enteroviruses. Phylogenetic analysis revealed three new types of caprine/ovine enterovirus based on the sequence identity of VP1, and provided novel evidence that caprine/ovine enteroviruses can be divided into six different genotypes/serotypes within EV-G.

## Figures and Tables

**Table 1:** The complete genome sequence identity of caprine/ovine enterovirus strains with reference strains of EV-G.

1	2	3	4	5	6	7	8	9	10	11		Virus Strains
	76.9	75.3	87.8	81.8	81.2	81.3	80.6	79.2	79.8	64.4	1	CEV-JL14
		75.1	76.7	76.2	76.3	76.4	75.0	75.5	75.6	63.9	2	TB4-OEV
			75.5	75.0	75.4	75.4	78.6	78.2	77.5	63.3	3	990/UK-NI
				79.9	79.2	79.3	78.3	78.3	77.7	64.5	4	JL-LS174
					91.9	92.0	81.0	79.0	79.4	64.5	5	JL-LS127
						98.8	80.3	78.3	78.7	64.4	6	JL-LS165
							80.4	78.3	78.7	64.3	7	JL-LS34
								83.5	82.5	64.6	8	NMG-F37
									86.0	64.8	9	NX-DR26
										64.5	10	SD-S68
											11	SD-S67

**Table 2:** Percentage identities of VP1 amino acid sequences of caprine/ovine enterovirus strains compared with reference strains of EV-G.

1	2	3	4	5	6	7	8	9	10	11		Virus Strains
	68.7	61.7	95.8	67.3	67.3	66.9	64.2	66.3	65.6	52.0	1	CEV-JL14
		61.0	69.7	74.3	73.6	73.9	62.8	66.3	65.2	52.7	2	TB4-OEV
			60.6	59.9	59.9	59.3	77.4	75.3	74.9	50.9	3	990/UK-NI
				66.9	66.9	66.5	64.2	67.0	66.3	51.6	4	JL-LS174
					99.3	98.6	61.3	62.1	61.7	49.1	5	JL-LS127
						97.6	61.3	62.1	61.7	48.7	6	JL-LS165
							60.6	61.3	61.0	48.7	7	JL-LS34
								78.8	79.2	52.0	8	NMG-F37
									95.1	53.5	9	NX-DR26
										52.0	10	SD-S68
											11	SD-S67

## Data Availability

The reference sequences used for phylogenetic analysis included GenBank accession numbers MG958646.1, KP240936.1, NC_021220.1, NC_034267.1, AY896765.1, AY896767.1, KF748290.1, AF123432.1, AF414373.2, KM402020.1, KM402021.1, KT581587.1, HQ702854.1, JQ818253.1, JQ277724.1, KM851231.1, HQ738303.1, HQ738302.1, JN807387.1, MK639928.1, NC_004441.1, AF363455.1, KP982873.1, KY761948.1, KT265911.2, KT265961.2, KJ156451.1, KT265900.1, KT265903.1, KT265909.1, KT265941.1, MF113370.1, NC_038309.1, NC_038310.1, NC_038989.1, KX156159.1, NC_029905.1, NC_038311.1, FJ445112.1, EF186077.2, and MF113372.1.

## Supplementary Materials

The following supporting information can be downloaded at: https://www.mdpi.com/article/10.3390/v14051051/s1. Table S1: Primer sequences to amplify full-length sequences; Table S2: Reference strains for enterovirus; Table S3: Basic information for the caprine/ovine enterovirus isolates; Table S4: The complete genome sequence of the newly identified viral strains; Table S5: The p-distance of VP1 amino acid sequences between EV-G, HY12, and SD-S67; Figure S1: Recombination analyses of JL-LS127 with EV-G. Each curve is a comparison between the genome being analyzed and a reference genome. The horizontal bar above the curves is an illustration of the enterovirus genome. Each point plotted is the percentage identity within a sliding window 200 nt wide centered on the position plotted, with a step size of 20 nt between points; Figure S2: Recombination analyses of JL-LS165 with EV-G. Each curve is a comparison between the genome being analyzed and a reference genome. The horizontal bar above the curves is an illustration of the enterovirus genome. Each point plotted is the percentage identity within a sliding window 200 nt wide centered on the position plotted, with a step size of 20 nt between points; Figure S3: Recombination analyses of JL-LS174 with EV-G. Each curve is a comparison between the genome being analyzed and a reference genome. The horizontal bar above the curves is an illustration of the enterovirus genome. Each point plotted is the percentage identity within a sliding window 200 nt wide centered on the position plotted, with a step size of 20 nt between points; Figure S4: Recombination analyses of NX-DR26 with EV-G. Each curve is a comparison between the genome being analyzed and a reference genome. The horizontal bar above the curves is an illustration of the enterovirus genome. Each point plotted is the percentage identity within a sliding window 200 nt wide centered on the position plotted, with a step size of 20 nt between points; Figure S5: Recombination analyses of SD-S68 with EV-G. Each curve is a comparison between the genome being analyzed and a reference genome. The horizontal bar above the curves is an illustration of the enterovirus genome. Each point plotted is the percentage identity within a sliding window 200 nt wide centered on the position plotted, with a step size of 20 nt between points; Figure S6: Recombination analyses of NMG-F37 with EV-G. Each curve is a comparison between the genome being analyzed and a reference genome. The horizontal bar above the curves is an illustration of the enterovirus genome. Each point plotted is the percentage identity within a sliding window 200 nt wide centered on the position plotted, with a step size of 20 nt between points.
