# Supplementary material for: Molecular Analysis of Caprine Enterovirus Circulating in China during 2016–2021: Evolutionary Significance
Source: Viruses. 2022 May 15;14(5):1051. doi: 10.3390/v14051051 (PMC9143109; doi:10.3390/v14051051)
Supplement: Supplementary file 1 [file viruses-14-01051-s001.zip › Fig S2.pdf]

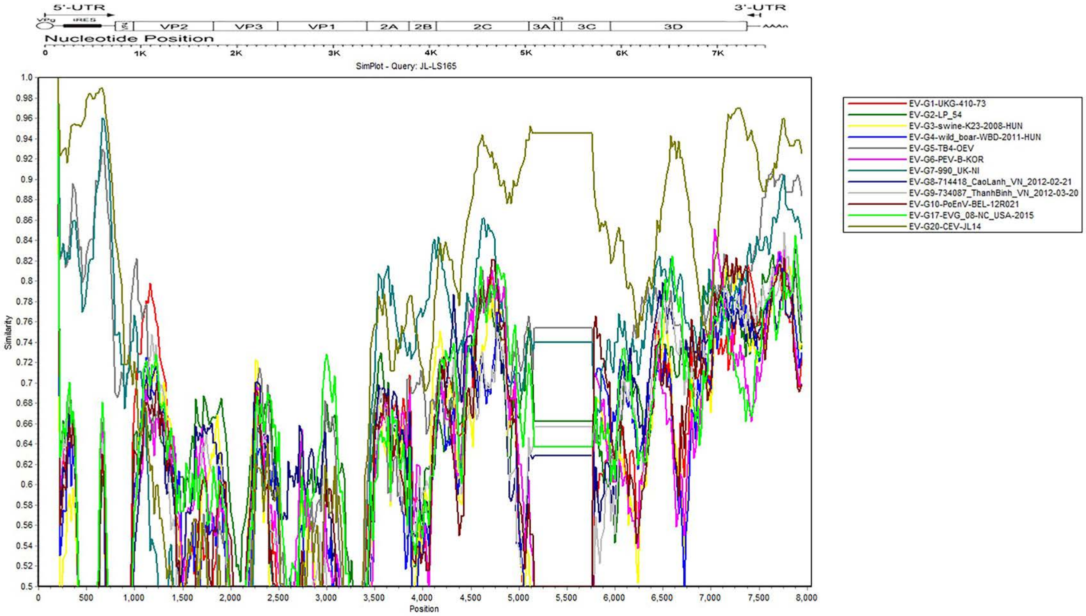

5'-UTR
3'-UTR
VPg
IRES
VP4
VP2
VP3
VP1
2A
2B
2C
3A
3B
3C
3D
AAAn
Nucleotide Position
0
1K
2K
3K
4K
5K
6K
7K
SimPlot - Query: JL-LS165
Similarity
Position
EV-G1-UKG-410-73
EV-G2-LP_54
EV-G3-swine-K23-2008-HUN
EV-G4-wild_boar-WBD-2011-HUN
EV-G5-TB4-OEV
EV-G6-PEV-B-KOR
EV-G7-990_UK-NI
EV-G8-714418_CaoLanh_VN_2012-02-21
EV-G9-734087_ThanhBinh_VN_2012-03-20
EV-G10-PoEnV-BEL-12R021
EV-G17-EVG_08-NC_USA-2015
EV-G20-CEV-JL14
