# Supplementary material for: Molecular Analysis of Caprine Enterovirus Circulating in China during 2016–2021: Evolutionary Significance
Source: Viruses. 2022 May 15;14(5):1051. doi: 10.3390/v14051051 (PMC9143109; doi:10.3390/v14051051)
Supplement: Supplementary file 1 [file viruses-14-01051-s001.zip › Table S1.pdf]

Table S1 Primer sequences to amplify full-length sequences

Name	Sequences	Position
CEV-1-F	ACGTGGCGGTAGTGCTTTGGTTTG	41 ~ 64
CEV-1-R	GCTTGGGTGGCGAGGTGTCTGTT	3393 ~ 3415
CEV-2-F	GTGGTAGCTCTGAGTGAT	385 ~ 402
CEV-2-R	TCGTTGCTTATTCTTGAACT	4423 ~ 4442
CEV-3-F	GTGGTAGCTCTGAGTGAT	385 ~ 402
CEV-3-R	GGATTTGCAATAATAGAC	3516 ~ 3533
CEV-4-F	GCGCGGTGGCACAGTTAGG	3160 ~ 3178
CEV-4-R	CACGCCCGACTGGAACTGAGC	7264 ~ 7284
CEV-5-F	GGTTACATACAGTTCAAGAA	3413 ~ 3432
CEV-5-R	ATAACCGGATGTATGAGGA	7111 ~ 7129
CEV-6-F	TTTAAAACAGCCTGGGGGTTGT	1 ~ 22
CEV-6-R	TAGGCGCTTCTCTAGTTAC	489 ~ 507
