# Supplementary material for: Molecular Analysis of Caprine Enterovirus Circulating in China during 2016–2021: Evolutionary Significance
Source: Viruses. 2022 May 15;14(5):1051. doi: 10.3390/v14051051 (PMC9143109; doi:10.3390/v14051051)
Supplement: Supplementary file 1 [file viruses-14-01051-s001.zip › Table S2.pdf]

Table S2 Reference strains for enterovirus

Strains	Sequence	Accession No	Species	Host
990/UK-NI	Complete	MG958646.1	G	sheep
Beijing-R0132	Complete	KP240936.1	D	Homo sapiens
BEV-261	Complete	NC_021220.1	F	Bovine
CEV-JL14	Complete	NC_034267.1	G	Capra hircus
EV7-15936-01	Complete	AY896765.1	B	sewage
EV30-8477-98	Complete	AY896767.1	B	Human with meningitis
HY12	Complete	KF748290.1	E	Bovine
K2577	Complete	AF123432.1	E	Bovine
N203	Complete	AF414373.2	J	simian
swine/K23/2008/HUN	Complete	HQ702854.1	G	Sus scrofa
PEV-B-KOR	Complete	JQ818253.1	G	Pig
S0098b/CA16/2013/CHN	Complete	KM402020.1	A	Homo sapiens
S0102b/EV71/2013/CHN	Complete	KM402021.1	A	Homo sapiens
Sev-nj1	Complete	KT581587.1	J	primate
TB4-OEV	Complete	JQ277724.1	G	Ovis aries
US/KY/14-18953	Complete	KM851231.1	D	Homo sapiens
V2-Tol.1	Complete	HQ738303.1	C	Homo sapiens
V3-Tul.7	Complete	HQ738302.1	C	Homo sapiens
wild boar/WBD/2011/HUN	Complete	JN807387.1	G	wild boar
SD-S67	Complete	MK639928.1	F	goat
UKG/410/73	Complete	NC_004441.1	G	Pig
LP 54	Complete	AF363455.1	G	Pig
PoEnV-BEL-12R021	Complete	KP982873.1	G	Sus scrofa
EVG 08/NC	VP1	KY761948.1	G	Sus scrofa domesticus
714418	VP1	KT265911.2	G	Pig
734087	VP1	KT265961.2	G	Pig
744257	VP1	KJ156451.1	G	Pig
714222	VP1	KT265900.1	G	Pig
714270	VP1	KT265903.1	G	Pig
714405	VP1	KT265909.1	G	Pig

724307	VP1	KT265941.1	G	Pig
GER/F26-2	VP1	MF113370.1	G	Sus scrofa domesticus
1715 UWB	Complete	NC_038309.1	H	Simian
19CC	Complete	NC_038310.1	I	Dromedary
rodent/Mc/PicoV/Tibet2015	Complete	KX156159.1	K	Rodent
SEV-gx	Complete	NC_029905.1	L	Macaca mulatta
ATCC VR-1559	Complete	NC_038311.1	Rhinovirus A	Human
ATCC VR-485	Complete	FJ445112.1	Rhinovirus B	Human
HRV-QPM	Complete	EF186077.2	Rhinovirus C	Human
rodent/Ee/PicoV/NX2015	Complete	NC_038989.1	K	rodent
GER/F8-2	VP1	MF113372.1	G	Sus scrofa domesticus
