# Supplementary material for: Molecular Analysis of Caprine Enterovirus Circulating in China during 2016–2021: Evolutionary Significance
Source: Viruses. 2022 May 15;14(5):1051. doi: 10.3390/v14051051 (PMC9143109; doi:10.3390/v14051051)
Supplement: Supplementary file 1 [file viruses-14-01051-s001.zip › Table S3.pdf]

Table S3 Basic information for the caprine/ovine enterovirus isolates

Isolate name	Origin	Isolation Year	Host	Isolation source	Clinical signs	Available sequence	$TCID_{50}$/0.1mL
JL-LS34	Jilin	2017	Goat	feces	Severe diarrhea	Complete genome	$10^{6.5}$
JL-LS127	Jilin	2017	Goat	feces	Severe diarrhea	Complete genome	$10^{6.33}$
JL-LS165	Jilin	2017	Goat	feces	Severe diarrhea	Complete genome	$10^{4.5}$
JL-LS174	Jilin	2017	Goat	feces	Severe diarrhea	Complete genome	10^{3}
SD-68	Shandong	2018	Goat	feces	soft feces	Complete genome	10^{6}
NMG-F37	Inner Mongolia	2016	Goat	feces	soft feces	Complete genome	$10^{4.23}$
NX-DR26	Ningxia	2018	Sheep	feces	soft feces	Complete genome	10^{3}
XJ-259	Xinjiang	2021	Sheep	feces	soft feces	5'UTR	$10^{4.8}$
XJ-274	Xinjiang	2021	Goat	feces	Severe diarrhea	5'UTR	10^{5}
XJ-287	Xinjiang	2021	Sheep	feces	soft feces	5'UTR	$10^{5.5}$
HeN-T3-12	Henan	2019	Goat	feces	soft feces	5'UTR	$10^{5.2}$
HeN-D1-37	Henan	2019	Goat	feces	soft feces	5'UTR	$10^{3.8}$
HeN-D2-57	Henan	2019	Goat	feces	soft feces	5'UTR	$10^{4.7}$
