# Supplementary material for: Molecular Analysis of Caprine Enterovirus Circulating in China during 2016–2021: Evolutionary Significance
Source: Viruses. 2022 May 15;14(5):1051. doi: 10.3390/v14051051 (PMC9143109; doi:10.3390/v14051051)
Supplement: Supplementary file 1 [file viruses-14-01051-s001.zip › Table S4.pdf]

Table S4 The complete genome sequence of the newly identified virus strains

Strains	Complete genome (bp)	5'UTR (bp)	ORF (bp)	3'UTR (bp)	Accession No
JL-LS34	7447	821	6519	107	MN598033
JL-LS127	7467	820	6519	128	MN598034
JL-LS165	7454	820	6519	115	MN598035
JL-LS174	7453	822	6519	112	MN598036
SD-S68	7465	821	6516	128	MN598040
NMG-F37	7469	819	6513	137	MN598041
NX-DR26	7463	820	6516	127	MN598038
