# Supplementary material for: Molecular Analysis of Caprine Enterovirus Circulating in China during 2016–2021: Evolutionary Significance
Source: Viruses. 2022 May 15;14(5):1051. doi: 10.3390/v14051051 (PMC9143109; doi:10.3390/v14051051)
Supplement: Supplementary file 1 [file viruses-14-01051-s001.zip › Table S5.pdf]

Table S5 The p-distance of VP1 amino acid sequence with EV-G, HY12, and SD-S67

		1	2	3	4	5	6	7	8	9	10	11	12	13	14	15	16	17	18	19	20	21	22	23	24	25	26	2[illegible]
1	JL-LS34-VP1																											
2	JL-LS127-VP1	0.02																										
3	JL-LS165-VP1	0.02	0.01																									
4	JL-LS174-VP1	0.30	0.30	0.30																								
5	NMG-F37-VP1	0.37	0.36	0.36	0.33																							
6	NX-DR26-VP1	0.36	0.36	0.36	0.30	0.20																						
7	SD-S68-VP1	0.37	0.36	0.36	0.30	0.19	0.04																					
8	EV-G20-CEV-JL14-VP1	0.30	0.30	0.30	0.04	0.32	0.30	0.31																				
9	EV-G5-TB4-OEV-VP1	0.24	0.23	0.24	0.25	0.34	0.31	0.32	0.26																			
10	EV-G7-990 UK-NI-VP1	0.39	0.38	0.38	0.36	0.21	0.23	0.23	0.35	0.35																		
11	EV-G17-EVG 08-NC USA-2015-VP1	0.20	0.19	0.19	0.29	0.37	0.34	0.34	0.29	0.23	0.39																	
12	EV-G1-UKG-410-73-VP1	0.38	0.38	0.38	0.40	0.47	0.47	0.46	0.40	0.39	0.47	0.37																
13	EV-G10-PoEnV-BEL-12R021-VP1	0.33	0.33	0.33	0.34	0.41	0.39	0.38	0.35	0.34	0.42	0.30	0.32															
14	EV-G11-744257-VP1	0.30	0.30	0.30	0.33	0.36	0.35	0.35	0.32	0.31	0.35	0.26	0.28	0.25														
15	EV-G12-714222 CaoLanh VN 2012-02-16-VP1	0.30	0.29	0.30	0.31	0.37	0.34	0.34	0.31	0.30	0.37	0.28	0.31	0.24	0.21													
16	EV-G13-714270 CaoLanh VN 2012-02-16-VP1	0.34	0.34	0.34	0.36	0.39	0.35	0.36	0.36	0.33	0.40	0.32	0.37	0.28	0.26	0.23												
17	EV-G14-714405 CaoLanh VN 2012-02-21-VP1	0.33	0.32	0.33	0.35	0.41	0.40	0.39	0.34	0.33	0.42	0.30	0.27	0.26	0.19	0.23	0.28											
18	EV-G15-724307 ChauThanh VN 2012-03-14-VP1	0.32	0.32	0.32	0.35	0.41	0.38	0.38	0.36	0.36	0.43	0.34	0.33	0.22	0.26	0.26	0.29	0.27										

19	EV-G16-BS14-173H2 DakLak VN 2014-VP1	0.34	0.34	0.34	0.34	0.42	0.39	0.39	0.35	0.32	0.44	0.30	0.31	0.28	0.26	0.28	0.27	0.24	0.30											
20	EV-G18-GER F26-2 23-12-2013-VP1	0.21	0.19	0.20	0.31	0.38	0.34	0.34	0.31	0.25	0.37	0.16	0.36	0.33	0.27	0.26	0.32	0.32	0.33	0.31										
21	EV-G19-GER F8-2 04-02-2013-VP1	0.29	0.28	0.29	0.31	0.39	0.37	0.36	0.31	0.30	0.40	0.26	0.31	0.26	0.23	0.17	0.24	0.28	0.25	0.29	0.26									
22	EV-G2-LP 54-VP1	0.21	0.21	0.21	0.30	0.35	0.34	0.33	0.29	0.23	0.36	0.19	0.36	0.33	0.27	0.30	0.33	0.30	0.32	0.32	0.19	0.28								
23	EV-G3-swine-K23-2008-HUN-VP1	0.36	0.36	0.36	0.39	0.41	0.42	0.41	0.38	0.39	0.44	0.35	0.36	0.24	0.26	0.27	0.28	0.26	0.20	0.31	0.38	0.29	0.37							
24	EV-G4-wild boar-WBD-2011-HUN-VP1	0.31	0.30	0.31	0.35	0.40	0.37	0.37	0.36	0.29	0.41	0.30	0.33	0.26	0.25	0.29	0.29	0.28	0.26	0.27	0.30	0.26	0.31	0.28						
25	EV-G8-714418 CaoLanh VN 2012-02-21-VP1	0.29	0.28	0.28	0.33	0.33	0.32	0.32	0.33	0.31	0.36	0.26	0.36	0.33	0.26	0.28	0.30	0.30	0.36	0.34	0.24	0.30	0.28	0.37	0.34					
26	EV-G6-PEV-B-KOR-VP1	0.30	0.30	0.30	0.38	0.38	0.38	0.38	0.37	0.32	0.39	0.31	0.32	0.25	0.23	0.25	0.27	0.26	0.24	0.27	0.30	0.26	0.30	0.27	0.15	0.32				
27	EV-G9-734087 ThanhBinh VN 2012-03-20-VP1	0.34	0.34	0.34	0.33	0.42	0.39	0.38	0.33	0.35	0.42	0.33	0.33	0.18	0.23	0.23	0.26	0.26	0.15	0.28	0.33	0.26	0.36	0.17	0.27	0.36	0.25			
28	SD-S67-VP1	0.49	0.49	0.49	0.47	0.47	0.45	0.47	0.47	0.45	0.49	0.46	0.50	0.47	0.45	0.46	0.45	0.45	0.49	0.50	0.49	0.48	0.48	0.47	0.46	0.46	0.48	0.4[illegible]		
29	HY12-VP1	0.50	0.50	0.50	0.49	0.51	0.48	0.49	0.49	0.49	0.51	0.50	0.53	0.51	0.47	0.50	0.49	0.50	0.49	0.52	0.50	0.51	0.50	0.51	0.49	0.49	0.50	0.5[illegible]		

Blue: the p-distance between JL-LS34, JL-LS127 and JL-LS165; Orange: the p-distance between NX-DR26 and SD-S68
